# Preliminary Insights in Sensory Profile of Sweet Cherries

**DOI:** 10.3390/foods10030612

**Published:** 2021-03-13

**Authors:** Vânia Silva, Sandra Pereira, Alice Vilela, Eunice Bacelar, Francisco Guedes, Carlos Ribeiro, Ana Paula Silva, Berta Gonçalves

**Affiliations:** 1 Centre for the Research and Technology of Agro-Environmental and Biological Sciences (CITAB), University of Trás-os-Montes and Alto Douro (UTAD), Quinta de Prados, 5000-801 Vila Real, Portugal; sirp@utad.pt (S.P.); areale@utad.pt (E.B.); asilva@utad.pt (A.P.S.); bertag@utad.pt (B.G.); 2 Chemistry Research Centre (CQ-VR), Department of Biology and Environment, University of Trás-os-Montes and Alto Douro (UTAD), Quinta de Prados, 5000-801 Vila Real, Portugal; avimoura@utad.pt; 3 Cermouros-Cerejas de São Martinho de Mouros, Lda., Quinta da Ribeira, Bulhos, 4660-210 Resende, Portugal; fjmguedes@cermouros.pt; 4 Department of Agronomy, University of Trás-os-Montes and Alto Douro (UTAD), Quinta de Prados, 5000-801 Vila Real, Portugal; cribeiro@utad.pt

**Keywords:** *Prunus avium* L., cv. Burlat, cv. Van, cherry preferences, fruit quality traits, physicochemical characteristics, sensory profile

## Abstract

Sweet cherry (*Prunus avium* L.) is a fruit appreciated by consumers for its well-known physical and sensory characteristics and its health benefits. Being an extremely perishable fruit, it is important to know the unique attributes of the cultivars to develop cultivation or postharvest strategies that can enhance their quality. This study aimed to understand the influence of physicochemical characteristics of two sweet cherry cultivars, Burlat and Van, on the food quality perception. Several parameters (weight, dimensions, soluble solids content (SSC), pH, titratable acidity (TA), colour, and texture) were measured and correlated with sensory data. Results showed that cv. Van presented heavier and firmer fruits with high sugar content. In turn, cv. Burlat showed higher pH, lower TA, and presented redder and brightest fruits. The principal component analysis revealed an evident separation between cultivars. Van cherries stood out for their sensory parameters and were classified as more acidic, bitter, and astringent, and presented a firmer texture. Contrarily, Burlat cherries were distinguished as being more flavourful, succulent, sweeter, and more uniform in terms of visual and colour parameters. The results of the sensory analysis suggested that perceived quality does not always depend on and/or recognize the quality parameters inherent to the physicochemical characteristics of each cultivar.

## 1. Introduction

Sweet cherry production around the world has been growing in the last years. The worldwide estimation of sweet cherry production, in 2018, was 2,563,134 t in a harvested area of 441,953 ha. Asia represented the largest producer with 44.6% of world production, followed by Europe with 33.7%, and the Americas with 19.8% [1].

Sweet cherry is one of the most popular and appreciated early Spring/Summer fruits; thus, it is extremely important to obtain high-quality fruits that meet the consumer’s expectations [2,3,4]. Because it is a very perishable fruit that is consumed essentially in a fresh state, it is sometimes difficult to maintain these demanding levels [3,4,5]. To achieve excellent products, it is essential to know in advance the characteristics inherent to each cultivar, as well as how these characteristics are perceived by the consumer. Thus, strategies that can enhance the quality should be developed, such as the appropriate selection of the combination cultivar x rootstock, choosing the optimum harvest date, and the application of pre-and post-harvest compounds, namely bio stimulants and bio fungicides, among others [3,4,5,6,7].

According to FAO/WHO (Food and Agriculture Organization of the United Nations, Rome, Italy/World Health Organization, Geneva, Switzerland) [8], food quality covers all the characteristics that influence the appreciation of a product by the consumer. This includes negative characteristics (dirt, deterioration, discoloration, abnormal odours, etc.) and positive attributes (colour, texture, flavour, origin, and type of food processing, etc.).

The term quality has been defined in different ways, over time. However, there seems to be a consensus that quality can be distinguished in objective (physical attributes introduced to the product) and subjective (quality perceived by consumers) [9]. This last one can be divided into two approaches: a holistic method in which quality encompasses all desirable properties that are perceived in the product and, an excellence approach that suggests that parts may not constitute the whole. This suggests that products may have properties appreciated and valued by consumers, but that they are not always seen as a quality factor [9]. Thus, it is important to know the preferences of consumers, that is, their perceived quality. The appreciation of food products is connected with the senses, mainly with texture, aroma, and flavour [10]. The process that establishes consumer acceptability for a product is called a sensory evaluation test [11]. The sensory evaluation uses humans to measure sensory perceptions and understand their acceptance of food and taste. The information from the sensory evaluation is useful in marketing decisions because it allows us to identify, quantify, and idealise the main drivers for the acceptance of a product. Nowadays, any sensory program recognizes this information as an elementary resource [10].

As the cherry fruit is particularly sensitive and perishable, its quality is affected and tends to decrease along each step of the supply chain. Therefore, high care levels must be taken besides good decisions and good practices, that should begin at the orchard, to achieve premium quality fruit for distribution in the fresh market [3,5,6,12,13,14], thereby satisfying the consumer. Physical damages should be avoided because, in addition to being considered defects, they accelerate water loss, influencing its deterioration, contamination, and, consequently, the shelf life [4,5,15].

Based on consumer acceptance, the quality of sweet cherries is defined by several attributes, such as visual appearance (size, bright red colour, and green peduncle colour), sweetness, firmness, and flavour [16,17,18]. Colour is defined as the main indicator of the fresh cherries quality, by several authors [17,19,20,21]. Additionally, Girard and Kopp [22] consider firmness as an important textural attribute often used to assess fruit quality. The requirements of international markets on cherry quality, especially the fruit dimension, have increased notably [23] being a decisive parameter of fruit value [24]. From the consumer’s point of view, an ‘ideal’ cherry should be large, dark, and sweet, with a shiny appearance being the attributes that influence the purchase decision [25,26]. A good balance between sugars (sweetness) and fruit acids (sourness), combined with the absence of defects such as cracks, stings of birds/insects, rot, among others, are also important quality criteria [3,11,27] and function as critical factors in consumer acceptance [20]. Also important is the chemical composition of sweet cherries which has a great influence on the sensory quality of the fruits [28]. These fruits are a source of bioactive compounds, such as, natural polyphenolic compounds, that enhance health benefits (health promoters), revealing an anti-cancer, anti-inflammatory, and antioxidant power, among others [3,29,30,31,32]. Therefore, it is important to evaluate their impact on consumer acceptance, trying to achieve the optimal combination of taste and health-promoting compounds [33].

To better understand how the perception of the sensory quality of sweet cherry can be influenced by the characteristics of each cultivar, chemical, textural, and sensory parameters were measured/evaluated, and correlated.

## 2. Materials and Methods

### 2.1. Experimental Design, Fruit, and Sampling

**Figure 1:**
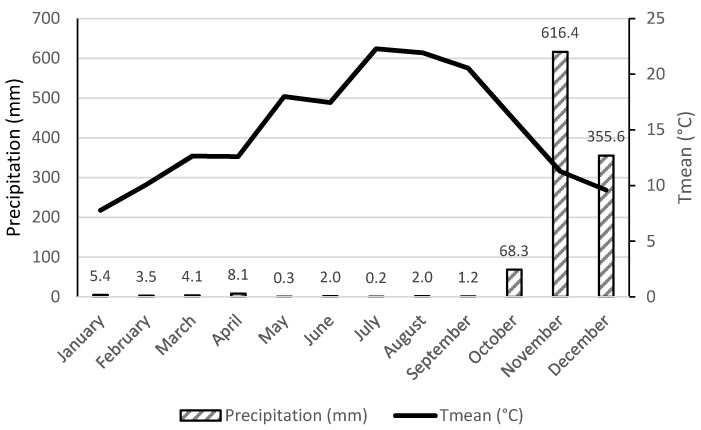
Weather conditions: precipitation (mm) and mean temperature (°C), in the study year (2019).

The trial took place in a commercial orchard that was 10 years old, located on a hillside in the region of Alufinha, Resende (Viseu District), Portugal (41°12′ N, 7°93′ W, altitude 149 m). The trees have a spacing of 3.0 m between lines and 2.5 m from each other on the line, trained in a vertical system. Weather data were recorded by a weather station located in São João da Fontoura, near the experimental site (Figure 1). The year 2019 was extremely hot and dry. The mean temperature ranged from 7.8 °C (January) to 22.3 °C (July) and rainfall values for the area were relatively low throughout the year with a peak of precipitation in the last 3 months (maximum in November with 616.4 mm).

For this trial, ten trees of each cultivar in the study, namely, cv. Burlat (an early cultivar) and cv. Van (a late cultivar), both grafted on ‘Colt’ rootstock, were selected. In these trees, about 1 kg of fruits was randomly chosen and carefully harvested, at the stage of commercial maturity (cv. Burlat on 8 May and cv. Van on 27 May 2019), and then transported to the laboratory, under refrigerated conditions. On the same day, after arriving at the laboratory, the respective routine analyses (weight, dimensions, soluble solids content (SSC), pH, titratable acidity (TA), colour, and texture), and sensory tests were immediately carried out.

### 2.2. Determination of Physicochemical Parameters of Sweet Cherry

#### 2.2.1. Weight and Dimensions

In a sample of 30 fruits, without defects, of each cultivar, the weight of each fruit was determined, using an electronic balance (PCD2200-2, Kern, Balingen, Germany, with an accuracy of 0.01 g). Additionally, measurements of their size (larger diameter, smaller diameter, and height) were carried out, using a digital caliper (Mitutoyo absolute digimatic, with 0.01 mm of precision).

#### 2.2.2. Chromatic Characteristics

Fruit colour was measured at both sides of these same 30 fruits with a colorimeter (CR-300, Minolta, Osaka, Japan) following the CIE (Commission International de l’Eclairage, Vienna, Austria) system of 1976, using the psychometric space (CIELAB). This system consistently correlates colour values with visual perception. Colours can be expressed in terms of hue (h°), lightness (L*), and saturation (chromatic coordinates a* and b*). L* can be read from 0 (opaque or black) to 100 (transparent or white), coordinate a* from red to green (+a indicates red and -a indicates green), co-ordinate b* from yellow to blue (+b indicates yellow and -b indicates blue). It can also be described using cylindrical coordinates being lightness (L*), hue (*h*°), and chroma (C*), related to the Munsell coordinates [34,35]. The hue = arctg (b*/a*), expressed the colour nuance [24], and values, presented in degrees, vary between 0° to 90° (red to yellow), 180° to 270° (green to bluish-green) [36]. The chroma (C*) of cherry fruits was expressed according to the formula: C* = (a*^2^ + b*^2^)^1/2^ [37,38,39]. Results were presented as the average of sixty values obtained for 30 fruits from each cultivar with the indication of standard deviation (SD).

#### 2.2.3. Epidermis Rupture Force and Flesh Firmness

The texture of the cherries was measured using a Texture Analyser (TA.XT.plus–Stable Micro Systems, Godalming, UK). For that, a load cell of 50 N and a cylindrical probe with 2.0 mm diameter, were used and the maximum force required to compress 5 mm of pulp at a speed of 1 mm s^−1^ was recorded.

The captured and analysed data (force, distance, and time) indicated the sample texture in terms of flesh firmness of pulp—FF (N mm^−1^) and the epidermis rupture force—ERF (N). Results were expressed as an average of thirty fruits with the indication of SD.

#### 2.2.4. Soluble Solids Content, pH, and Titratable Acidity

The same 30 fruits of each cultivar used for fruit biometry, colour, and texture were divided into three groups of ten and the pulp was separated from the pit and the peduncle. With an electric extractor (ZN350C70, Tefal Elea, Hong Kong, China) all the juice from the pulp of those ten fruits were extracted. Total soluble solids content—SSC (°Brix) was measured using a digital refractometer (PR-101, Atago, Japan), followed by pH measurement with a pH meter (3310 Jenway, UK). Immediately thereafter, 10 mL of juice was diluted in 10 mL of distilled water and automatic titration (using an automatic titrator—Schott Easy Titroline) of the solution with sodium hydroxide (NaOH, 0.1 mol L^−1^) until reaching pH 8.2, obtaining the titratable acidity (TA) (in g of citric acid by 100 g of fresh weight). Results were reported as the average of three replicates with the indication of SD.

### 2.3. Sweet Cherries Sensory Profile Evaluation

The sweet cherries sensory profile was evaluated by a trained panel of 12 judges, ranging from 35 to 50 years of age, belonging to the Department of Biology and Environment, School of Life and Environment Sciences, University of Trás-os-Montes and Alto Douro (DeBA/ECVA-UTAD), with high expertise in the evaluation of fruits and fruits-derived products and that had already participated in several scientific works [40,41,42,43,44]. Nevertheless, before the cherries-sample tasting sessions, a training session was performed for the familiarization of the panellists with the selected tasting sheet and cherries sensory attributes (Table 1), using standard commercial cherries available in the market at that time.

The sensory tests took place in a laboratory equipped for sensory analysis according to ISO 6658 [45]. Panellists evaluated a list of twelve cherry attributes (Table 1) adapted from Chauvin et al. [46]. The attributes were classified according to the intensity scale provided and scored from 1 (lowest intensity) to 5 (highest intensity) points [47,48]. Before the session started, the randomly coded samples were washed with distilled water and placed at room temperature for 2 h to obtain a temperature of about 18 °C. After, the samples were presented to the tasters, on a white Pyrex plate, two cherry fruits per cultivar, for each one. Between each tasting, as a flavour cutter, tasters were asked to drink a sip of water or bite on a low-salt cracker. In order not to affect the tasting performance, the tasters were non-smokers, did not wear any type of perfume, and one hour before the start of the tasting session they did not eat or drink anything.

### 2.4. Statistical Analysis

Statistical analysis was performed using Software SPSS V.25 (SPSS-IBM Corp., Armonk, NY, USA). Statistical differences were evaluated by one-way analysis of variance (ANOVA), followed by Tukey’s post hoc test (*p* < 0.05), establishing the cultivar effect. Sensory data were analysed by one-way ANOVA followed by the post hoc Duncan’s multiple range test (*p* < 0.05).

Principal Component Analysis (PCA) was performed using the Pearson correlation matrix after normalizing the data matrix for each attribute, using the XLSTAT software. PCA is a mathematical procedure that allows converting a set of observations of possibly correlated variables into a set of linearly uncorrelated variable values (principal components).

## 3. Results and Discussion

### 3.1. Determination of Physicochemical Properties of Sweet Cherry

Evaluating the physicochemical properties of both sweet cherry cultivars (Table 2), it was possible to verify that the fruits of cv. Van presented higher fresh weight (9.30 vs. 8.10 g) and size (27.89 vs. 26.49 mm) than cv. Burlat. Identical results were found by Faniadis et al. [49]. Consumers from European countries revealed a preference for fruits between 11 and 12 g [25], whereas consumers in the USA value sweetness over the fruit size [50]. In terms of anatomical parameters, Kappel et al. [25], found that Canadian consumers considered the 29 mm size as the ideal diameter for sweet cherries. In the present study, Van and Burlat cherries presented a diameter of 27–28 mm; thus, they were in the optimal range of values found as optimal for consumer’s purchase.

**Figure 2:**
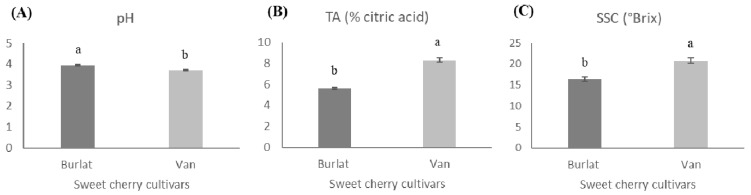
Sweetness and acidity of the two sweet cherry cultivars (Van and Burlat): (**A**) pH; (**B**) Titratable acidity (TA); (**C**) Total soluble solids content (SSC) of the two sweet cherry cultivars. The presented values resulted from the mean ± SD (*n* = 3). Statistically significant differences between cultivars, for each parameter, were represented by different letters (ANOVA-Tukey’s test, *p* < 0.05).

Considering that SSC varied between 11 and 25 °Brix in sweet cherry [31,51], in our study, fruits of cv. Van could be considered of the greatest sweetness (20.80 vs. 16.37 °Brix). On the other hand, the fruits of cv. Burlat had a higher pH value (3.94 vs. 3.70), and therefore, lower titratable acidity than cv. Van (5.60 vs. 8.30% citric acid), as displayed in Figure 2.

**Figure 3:**
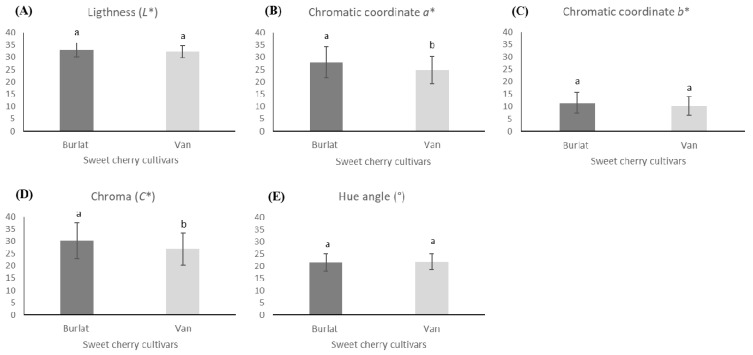
Chromatic parameters of the two sweet cherries cultivars (Van and Burlat): (**A**) Lightness (L*); (**B**) Chromatic coordinate a*; (**C**) Chromatic coordinate b*; (**D**) Chroma (C*) and (**E**) Hue angle (°). The presented values resulted from the mean ± SD (*n* = 60). Statistically significant differences between cultivars, for each parameter, were represented by different letters (ANOVA-Tukey’s test, *p* < 0.05).

The chromatic parameters of fruits, L*, a*, b*, chroma, and hue angle were shown in Figure 3. Overall, Burlat cherries had higher values than Van cherries in all parameters, but significant differences (*p* < 0.05) were only found in coordinate a* and chroma. In particular, Burlat had redder and brighter fruits (a* = 27.97; L* = 33.03) than cv. Van that showed significantly lower values (a* = 24.84; L* = 32.25). Identical results were found by Faniadis et al. [49]. This result was following other studies, which refer that chroma and hue angle of partially ripe cherries were always higher than in the ripe ones [38,52].

**Figure 4:**
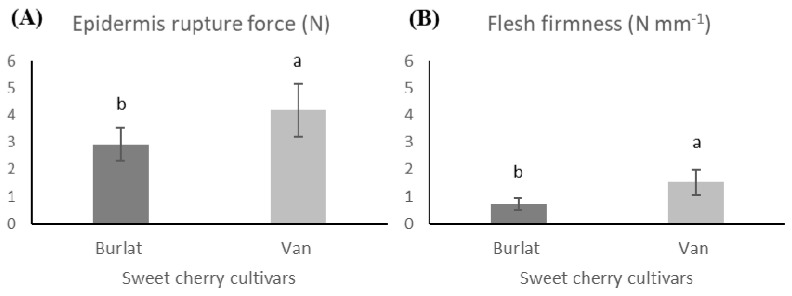
Texture of the two sweet cherry cultivars (Van and Burlat): (**A**) Epidermis rupture force—ERF (N) and (**B**) Flesh firmness—FF (N mm^−1^). The presented values resulted from the mean ± SD (*n* = 30). Statistically significant differences between cultivars, for each parameter, were represented by different letters (ANOVA-Tukey’s test, *p* < 0.05).

Usenik et al. [53] reported that the fruits of late cultivars were generally firmer than those of early cultivars, which was also observed in the present work, where cv. Van presented firmer fruits than cv. Burlat, offering higher resistance to rupture of the epidermis (Figure 4).

### 3.2. Sweet Cherries Sensory Profile Evaluation

**Figure 5:**
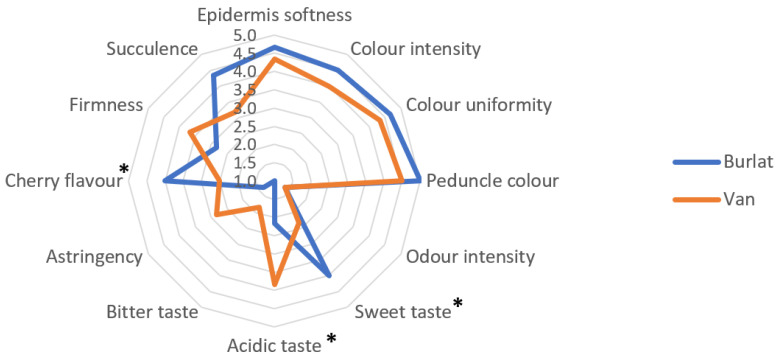
Spider plot of the sensory profile of the two sweet cherry cultivars (Van and Burlat). (*) represented significant differences between cultivars, according to Duncan’s test, *p* < 0.05. The absence of superscript indicated no significant differences.

The sensory analysis (Figure 5) performed by a trained panel of tasters showed a low number of significant differences between cultivars. In twelve attributes, the tasters highlighted significant differences in only three: cherry flavour, acidic taste, and sweet taste. According to the panel, the cv. Van was more acidic while cv. Burlat was sweeter and with a more intense cherry flavour. Other characteristics identified in routine analyses, such as firmness, colour, weight, and dimensions went unnoticed to tasters.

**Figure 6:**
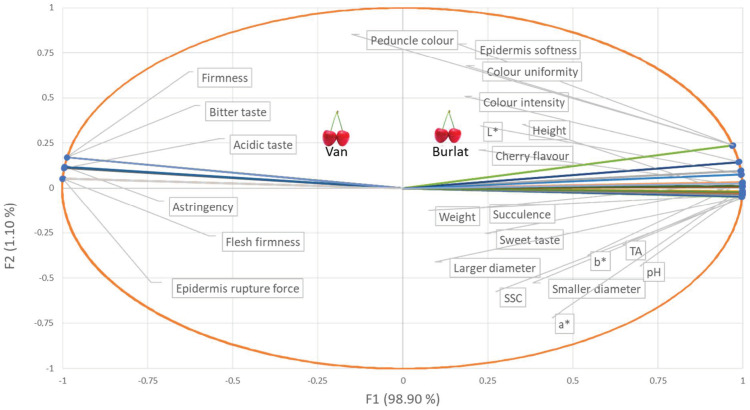
Principal component analysis (Pearson correlation) with factor 1 (F1) and factor 2 (F2) was obtained by analysis of sensory and analytical data of the two sweet cherry cultivars (Van and Burlat).

To understand how the evaluated parameters correlated with each other, a chemometric analysis was performed combining the chemical, textural, fruit weight, and dimensions, all together, with sensory data (Figure 6). The PCA based on the Pearson correlation matrix standardized the data. In this analysis, the two main components (PC1 and PC2) represented 100% of the total variance, with factor 1 (F1) reflecting the highest variability (98.90%). In the spatial projection of Figure 6, it was possible to visualize the distribution of the samples evaluated and the associated parameters. In this projection, the two cultivars were spatially separated. Van cherries were in the upper left PCA quadrant. The sensory and textural parameters that seemed to contribute to this separation were acid (sour) and bitter taste, astringency, and firmness (sensory parameters); epidermis rupture force, and flesh firmness (textural parameters). Regarding texture, as already mentioned, cv. Van stood out for presenting firmer fruits, which offered greater resistance to the rupture of the epidermis, corroborating the firmness perceived by the tasters. Thus, there was a positive correlation between instrumental parameters (FF and ERF) and the sensory attribute firmness. However, there was a negative correlation with the epidermis softness sensory attribute since the tasters attributed this characteristic to cv. Burlat. On the other hand, Burlat cherries were located in the upper right PCA quadrant, and were characterized by the sensory parameters: cherry flavour, succulence, sweet taste, colour intensity, peduncle colour, epidermis softness, and colour uniformity; and by the colour coordinates L* and b*. The colour coordinate a*, the chemical parameters pH, SSC, and TA, and the fruit weight and dimensions were in the lower right PCA quadrant, which means that they were correlated with each other, but they do not particularly differentiate the two varieties.

Odour intensity was an attribute that did not appear in the PCA once it did not present any variability. Thus, it was a characteristic of minor importance in terms of sensory quality, for both cultivars. Panellists referred that the cherry’s odour was not perceived until the first bite, so, it was not an attribute that may influence consumer’s purchase decisions, which could probably be related to the classification of the sweet cherry as a non-climacteric fruit.

Consequently, it was interesting to verify that the bitter and acid taste attributes of Van cherries were not correlated with the titratable acidity (TA), which was not in accordance to Da Conceição et al. [54] that correlated the acidic perception of citric, malic and tartaric acids (titratable acidity) with the full sensory perceived acidity. However, this PCA projection corroborated the sensory data, once the panellists perceived the Burlat cherries as being significantly sweeter despite their higher chemical acidity, expressed in the TA and pH values. Titratable acidity is an approximation of the total acidity of a solution since it does not measure all acids, it measures the bound and free hydrogen ions in the solution. Several studies have reported that, for a given pH, the increase in the sour taste intensity was directly related to the increase in TA [55,56]. Shallenberger [57] proposed that sour taste intensity was strictly related to the potential hydrogen ion concentration, but Pangborn [58], found no relation between pH, titratable acidity, and relative sour taste intensity of several organic acids. Acids can produce a sour taste but are also capable of inducing non-sour taste characteristics, such as bitterness, saltiness, and astringency [59,60]. As mentioned by Da Conceição et al. [54], the relationship between the intensity of sour and hydrogen ions, and the physiology of sour or acid taste perception is not that simple and was controversial.

Although the absence of statistical differences in sensory data, the sensory attribute firmness was correlated with the textural parameters flesh firmness and epidermis rupture. The same was found by other authors in fruits of small size, as grapes [61].

It was in the Van cultivar that the highest values of sugars (SSC) were found; however, this sweetness was not perceived by the tasters that classified the Burlat cherries as “sweeter”. The sweetness was one of the main drivers of the consumer´s preference. Given the complexity of sweetness evaluation, SSC was commonly used as an estimation of this parameter. Nevertheless, it has been demonstrated that SSC and sweet taste were poorly correlated [62].

In terms of a hedonic perspective, Burlat cherries presented attributes more related to the pleasantness of eating cherries like cherry flavour, succulence, sweet taste, colour intensity, and uniformity [63]. Moreover, the generality of the fully ripe cherry fruit had a bright shiny pale to deep red or even purple colour. Owing to their intense red colour, consumers related cherries with healthy beneficial and antioxidant properties [64]. On the other hand, Zheng et al. [50] also stated that consumers had diverse preferences for quality traits of sweet cherries. On average, consumers are willing to pay the highest prices for cherries that bigger, firmer, sweeter, and flavourish [50]. Of course, it must be considered that, usually, consumers cannot judge the cherries’ taste until after purchases are made.

## 4. Conclusions

Nowadays, ensuring a high production without affecting fruit quality is a big challenge for producers. Therefore, the producer must know the intrinsic quality characteristics of each cultivar, as well as the consumer’s quality preferences. Based on this knowledge, the producer will be able to develop cultivation strategies that can enhance the product, introducing measures to improve quality attributes that meet consumer expectations.

This study contributed to explaining how cherries’ sensory profile can be linked to fruit characteristics and estimated perceived quality. With this work, it was possible to conclude that cv. Van, being a late maturation cultivar, stood out for the presentation of bigger fruits, firmer and with a high content of soluble sugars, characteristics highly valued by the consumers. Sensorially it stood out for being perceived as more acidic and bitter than cv. Burlat, nevertheless, presented an agreeable firmness in the mouth, a sensory parameter also corroborated by textural analysis. In turn, cv. Burlat presented characteristics normally typical of earlier cultivars, e.g., higher pH value and lower TA, with a bright red colour and with a more intense perceived sweetness, despite their higher chemical acidity, expressed in the TA and pH values. However, as Burlat fruits were among the first to appear on the market, they reached higher prices, with consequently greater profitability for the producer.

In this sense, the physicochemical characteristics of the cherries of both cultivars proved that the quality of the fruit highly depends on the cultivar and suggests that the quality perceived by the consumer does not always meet the quality parameters inherent to the fruit. Therefore, we can infer that consumers may enjoy more the perceived sweetness and cherry flavour of cv. Burlat over the firmness and acidity of cv. Van, considering these attributes as essentials to the subjective quality of the fruit.

Moreover, the perception of visual attributes will lead the consumer to choose Burlat cherries more often Van cherries, especially if is not possible to taste the fruit before buying it.

In general, the used methods can be useful predictors of sensory evaluation.

In future studies, it would be interesting to increase the number of cultivars under study, as well as to extend the sensory tests to the common consumer. The calculation of the profitability of the cultivars should be carried out, since the differences in productivity and harvest time of these two cultivars are also important for the market and, consequently for the producer. Taking into account that the physicochemical characteristics of the fruits can be deeply influenced by the environment, it will be important to continue the study for at least another year.

## Figures and Tables

**Table 1:** Vocabulary adapted from Chauvin et al. [46], and reference standards used for descriptive sensory analysis of cherries.

Attribute	Description and a Reference Scale
Epidermis softness	Rough-1/Very smooth-5
Colour intensity	Light (almost white)-1/Dark red-5
Colour uniformity	Not uniform-1/Uniform-5
Peduncle colour	Brown-1/Green-5
Odour intensity	Slightly intense-1/Very intense-5
Sweet taste	Slightly sweet-1/Very sweet-5
Acidic taste	Low acid-1/High acid-5
Bitter taste	Slightly bitter-1/Very bitter-5
Astringency	Scarcely astringent-1/Very astringent-5
Cherry flavour	Not much flavour-1/Excellent flavour-5
Firmness	Force needed to crack the cherry Little-1/A lot-5
Succulence	Juice extracted from the cherry after chewing Little-1/A lot-5

**Table 2:** Fruit weight (g) and dimensions (mm) of the two sweet cherry cultivars (Van and Burlat).

Cultivars	Weight	Larger Diameter	Smaller Diameter	Height
	(g)	(mm)	(mm)	(mm)
Burlat	8.10 ± 0.59 ^b^	26.49 ± 0.86 ^b^	21.06 ± 0.74 ^b^	23.13 ± 1.01 ^b^
Van	9.30 ± 0.72 ^a^	27.89 ± 0.91 ^a^	22.94 ± 0.68 ^a^	23.82 ± 0.87 ^a^

The presented values resulted from the mean ± SD (*n* = 30). Statistically significant differences between cultivars, for each parameter, were represented by different letters (ANOVA-Tukey’s test, *p* < 0.05).
